# Mechanistic elucidation of *Rhizopus stolonifer*-fermented orange peel in enhancing *Saccharomyces cerevisiae* growth and multi-stress tolerance: process optimization, metabolomics, and pathway analysis

**DOI:** 10.1016/j.fochx.2026.103599

**Published:** 2026-02-05

**Authors:** Xinjie Wang, Tao Chen, Dan Yu, Jinping Li, Yang Zhang, Jianxing Yu, Jiayou Li

**Affiliations:** a College of Biological, Chemical Sciences and Engineering, Jiaxing University, Jiaxing 314001, China; b Suqian Product Quality Supervision and Testing Institute, Suqian 223800, China; c Qinghai Key Laboratory of Medicinal Animal and Plant Resources in Qinghai-Tibet Plateau, College of Life Science, Qinghai Normal University, Xining 810008, China

**Keywords:** *Saccharomyces cerevisiae*, Fermentation, Growth promoting, Yeast stress tolerance, Non-targeted omics analysis

## Abstract

*Saccharomyces cerevisiae*, a pivotal organism in ethanol fermentation, frequently encounters challenges in high-stress industrial settings due to ethanol toxicity, acidic conditions, and thermal stress. This study aimed to optimize the fermentation of orange peel to enhance bioactive compounds and evaluate its impact on the stress tolerance of *S. cerevisiae*. The optimized conditions led to an increase in the total flavonoid content of the fermented orange peel (FOP). Supplementation with FOP significantly enhanced yeast tolerance; viability exceeded that of the control under 20% ethanol, improved at pH 2.8, and cell counts increased after 24 h at 40 °C. Experiments conducted in a 15-l fermenter demonstrated that FOP significantly promotes sugar consumption and alcohol production. Further mechanistic analysis revealed that hesperidin and rutin in FOP both exhibit potential to enhance yeast tolerance. This approach not only offers a cost-effective method for valorizing orange waste but also enhances yeast productivity and biofuel yield.

## Highlights

• Optimized fermentation of orange peel enhances its bioactive components.
• Fermented orange peel promotes the growth of *Saccharomyces cerevisiae*.
• Fermented orange peel enhanced *S. cerevisiae* tolerance to ethanol, low pH, and heat stress significantly.
• Metabolomics shows that bioactive components in FOP enhance the physiological functions of yeast.
• Provides an innovative solution for the valorization of citrus waste and cost-effective biofuel production.

## 1 Introduction

*Saccharomyces cerevisiae* is the principal microorganism for industrial ethanol fermentation, converting sugars such as glucose into ethanol and CO_2_ via the Crabtree effect (Fan et al., 2025; Zheng et al., 2024). In high-gravity or lignocellulosic fermentations, yeast cells are exposed to multiple stresses, including high sugar concentrations, elevated ethanol levels (8–10%, v/v), low pH, and toxic by-products such as acetic acid and furfural, which collectively impair cell growth and fermentation efficiency (Wang et al., 2024; Zhang et al., 2024). Improving the stress tolerance of *S. cerevisiae* is therefore essential for achieving high ethanol titers, reducing energy consumption during distillation, and enhancing the economic feasibility of biofuel production (Xu et al., 2025).

Various strategies have been developed to enhance yeast robustness, including strain screening, adaptive evolution, genetic engineering, and metabolic optimization. Ethanol-gradient adaptive evolution and inhibitor-tolerant strain selection have successfully improved fermentation performance under high-gravity conditions (Xu et al., 2025). Process-level interventions, such as pH control and hydrolysate detoxification, can further alleviate metabolic stress (Sornlek et al., 2024). In addition, co-cultivation with non-*Saccharomyces* yeasts (e.g., *Metschnikowia pulcherrima*) has been reported to mitigate ethanol inhibition by redistributing carbon flux toward glycerol and organic acid formation (Carbon et al., 2023; Pandey & Negi, 2023). At the molecular level, engineering of glucose-sensing pathways (Snf3/Rgt2), energy regeneration systems, and stress-related genes, as well as modulation of vitamin biosynthesis, has been shown to improve tolerance to ethanol, low pH, and complex substrates (Berterame et al., 2018; Lane et al., 2023; van Aalst et al., 2023; Wu et al., 2022; Yatabe et al., 2023; Zhang et al., 2024).

In parallel with intracellular engineering, plant-derived bioactive substances and extracts have been demonstrated to extend the chronological lifespan of yeast and improve tolerance to ethanol, osmotic, and oxidative stresses (Alugoju et al., 2023; Wu, Wang, et al., 2024). Such strategies complement traditional nutritional supplementation, including amino acid fortification (Gobert et al., 2019; Wu et al., 2022), and are particularly attractive when based on agro-industrial byproducts. Indeed, solid-state fermentation of cereal byproducts such as wheat bran and oat bran has been shown to generate functional ingredients with enhanced antioxidant activity and growth-promoting effects on yeast, highlighting the potential of valorizing processing residues into functional fermentation auxiliaries (Călinoiu et al., 2019).

Orange peel (OP), an abundant citrus-processing byproduct, is rich in flavonoids, polysaccharides, and essential oils, which exhibit antioxidant, antimicrobial, anti-inflammatory, and cell-protective activities (Chen et al., 2017; Gavahian et al., 2018). Its antioxidant capacity is closely associated with cellular stress resistance, and OP extracts have been reported to significantly enhance radical-scavenging activity and intracellular redox homeostasis (Afzaal et al., 2022; Rodrigues & Pintado, 2024). From a sustainability perspective, converting OP into value-added functional products is consistent with circular economy principles and offers a low-cost alternative to synthetic fermentation additives (Martín et al., 2010).

Base on the bioactivity of OP, microbial fermentation provides an effective biotransformation strategy. Filamentous fungi are particularly suitable for the solid-state fermentation of plant byproducts owing to their versatile extracellular enzyme systems and strong substrate-degrading capacity (Abdelshafy et al., 2024). *Rhizopus stolonifer*, a food-grade fungus widely used in traditional fermentations, secretes multiple hydrolytic enzymes capable of modifying citrus peel cell wall components and releasing bound phytochemicals (Huynh et al., 2024; Razola-Díaz et al., 2024). Notably, in our previous study, *Rhizopus*-mediated fermentation of citrus by-products significantly increased antioxidant capacity and bioactive availability, providing a practical and experimental basis for selecting this strain in the present work. Accordingly, *R. stolonifer* was employed in this study to ferment OP and generate fermented orange peel (FOP) with enriched functional properties.

This study undertakes a comparative analysis of OP and its *Rhizopus*-fermented counterpart to investigate the impact of microbial fermentation on the chemical composition and functional potential of this botanical substrate. Both the unprocessed OP and FOP were incorporated into the *S. cerevisiae* culture, leading to a marked enhancement of the yeast's tolerance under extreme stress conditions. A subsequent metabolomic analysis was performed to elucidate the mechanisms underlying the improved tolerance of *S. cerevisiae* as induced by the fermented orange peel.

## 2 Materials and methods

### 2.1 Materials and instruments

*Rhizopus stolonifer* JP1118: This strain is deposited in the China Center for Type Culture Collection (CCTCC No. M20231702) and is specifically designated for the development of microbial resources in traditional fermented foods.

*Saccharomyces cerevisiae:* Obtained from Angie's Yeast Co., Ltd., Yichang, Hubei.

Orange peel (OP): Supplied by Huayu Food Co., Ltd., Xiangshan, Zhejiang.

Chemicals: Yeast extract, peptone, urea, anhydrous magnesium sulfate (MgSO₄), monopotassium phosphate (KH₂PO₄), ammonium sulfate [(NH₄)₂SO₄], ammonium chloride (NH₄Cl), ammonium carbonate [(NH₄)₂CO₃], diammonium oxalate, and methanol are all analytically grade.

Instruments: The chromatographic analysis was performed using an ACQUITY HPLC I-Class system coupled with a Xevo G3-XS QTOF mass spectrometer (Waters, USA).

### 2.2 Optimization of the production process of fermented orange peel (FOP)

#### 2.2.1 Preparation of fungal spore suspension

The preserved strain of *R. stolonifer* JP1118 was cultured on potato dextrose agar (PDA) plates and incubated at a temperature of 28 °C for a duration of 72 h. The spores were collected by washing the culture with 2 mL of sterile saline solution (0.85%, *w*/*v*) and transferring the resulting suspension into a 50 mL centrifuge tube. The suspension was vortexed for 3 min and subsequently filtered through sterile lens paper and quartz wool to eliminate hyphal debris. The filtrate was then diluted with sterile saline to obtain a spore concentration of 1 × 10^8^ spores/mL and stored at 4 °C for future use.

#### 2.2.2 Determination of flavonoid content

For both OP and FOP, an equal amount of dried powder (0.20 g, on a dry weight basis of the final products) was used for extraction and quantification. OP and FOP were subjected to drying at 60 °C for a duration of 48 h, followed by grinding and sieving through a 50-mesh sieve for subsequent use. The extraction and quantification of total flavonoids were conducted in accordance with the methodology outlined by R. Huang (Huang, 2019). Specifically, 0.20 g of OP or FOP powder was combined with 3.0 mL of methanol and underwent ultrasonic-assisted extraction for 30 min at 100 W and 50 °C. Post-extraction, the samples were allowed to equilibrate at room temperature for 12 h, after which they were centrifuged at 4000 rpm for 15 min to obtain the supernatants. This extraction procedure was repeated three times, and the resulting supernatants were combined. The combined supernatant was subsequently filtered and diluted with methanol to a final volume of 10 mL. The samples were then sealed and stored at −20 °C until further analysis. To prepare the sample for analysis, take 1 mL of the previously diluted extract and combine it with 5 mL of 90% diethylene glycol and 0.1 mL of 4 mol/L sodium hydroxide solution. Subsequently, add deionized water to adjust the total volume to 10 mL. Incubate the mixture in a water bath at 40 °C for 10 min, then allow it to cool to room temperature. Use a mixture without the sodium hydroxide solution as a blank control. Construct a standard curve using hesperidin concentrations ranging from 0 to 450 μg/mL, measured at a wavelength of 360 nm. The total flavonoid content of the samples is quantified as hesperidin equivalents. The linear regression equation for the hesperidin standard curve is determined to be y = 16.59x, with a correlation coefficient (R^2^) of 0.999.

#### 2.2.3 Single-factor optimization of FOP production process

The initial conditions for FOP production via solid-state fermentation were established as follows: orange peel powder was combined with ammonium dihydrogen phosphate at a concentration of 0.3% (w/w) and glucose at 3.0% (w/w), with the moisture content adjusted to 50% (w/w). The mixture was homogenized, and the pH was set to 6.0. A spore suspension was then inoculated into the substrate mixture at a volume of 4% (v/w). Fermentation was conducted under static conditions at 28 °C for a duration of 4 days. Drying was applied to inactivate microbial cells; therefore, FOP was treated as a non-viable fermented substrate. While fungal residues may remain as part of the matrix, no active *Rhizopus* growth or metabolism was involved during subsequent yeast experiments. To systematically assess the individual effects of key parameters on the total flavonoid content in FOP, a single-variable optimization method was utilized. In a series of sequential trials, each target variable (e.g., types of nitrogen sources) was independently varied across a series of predetermined gradients, while all other parameters were maintained at the baseline levels as described above.

#### 2.2.4 Response surface methodology for optimization of FOP production conditions

The Plackett-Burman design, as presented in Table 1, was utilized to identify the critical variables affecting flavonoid content in FOP, based on initial single-factor optimization findings. Three parameters-moisture content (A), fermentation time (B), and NH_4_H_2_PO_4_ dosage (C) were identified as key variables. Subsequently, a three-factor, three-level Box-Behnken RSM design was formulated, incorporating these significant factors into 17 experimental trials. Both the Plackett-Burman screening and RSM optimization were executed using Design-Expert version 13.0. The factor levels for RSM optimization of flavonoid extraction were outlined in Table 2. Ultimately, FOP samples were prepared under the optimized fermentation conditions.

**Table 1:** Levels of factors for Plackett-Burman analysis.

Level	A Fermentation time/days	B Inoculation volume/%	C Carbon source dosage/%	D NH_4_H_2_PO_4_ dosage/%	E Moisture content/%	F Fermentation temperature/°C
−1	6	2	3	0.20	50	24
1	10	6	5	0.40	70	32

**Table 2:** Levels of factors for FOP response surface analysis.

Level	A Moisture content/%	B Fermentation time/days	C Nitrogen source dosage/%
−1	40	6	0.30
0	50	7	0.35
1	60	8	0.40

### 2.3 Influence of orange peel extracts on the growth and stress tolerance of *S. cerevisiae*

Preparation of Primary Inoculum (Culture A): A lyophilized powder of *S. cerevisiae* (1.0 g) was reconstituted in 100 mL of a sterile glucose solution (2%, *w*/*v*) and incubated at 38 °C with agitation at 220 rpm for 30 min in an Erlenmeyer flask, as described in our previous work (Ma et al., 2023). The resulting Culture A was subsequently streaked onto optimized YPD agar plates and incubated for 36 h to produce secondary-activated yeast colonies. These colonies were harvested using sterile water and adjusted to a final concentration of 1 × 10^8^ spores/mL, thereby generating the working inoculum (Culture B).

In the experimental analysis of effects, YPD liquid medium was augmented with either 2 g/L of FOP or OP (added on an equal mass basis, both applied as dried and non-viable powders), followed by inoculation with 1% (v/v) of Culture B. The cultures were incubated statically at 28 °C for a duration of 30 h. Yeast growth dynamics were monitored bi-hourly through viable cell counting, and the specific growth rate (μ) was calculated to assess the influence of FOP and OP on yeast proliferation.

To evaluate the protective effects of FOP and OP on the stress tolerance of *S. cerevisiae*, three distinct stress conditions were established: thermal stress at 40 °C, ethanol-induced stress with concentrations ranging from 10% to 30%, and acidic stress with pH levels ranging from 2.0 to 3.6. Yeast viability under each stress condition was quantitatively assessed and compared between the FOP- and OP-supplemented groups to determine their respective effects as supplemented fermentation-derived products.

To systematically assess the stress-mitigating effects of FOP and OP on *S. cerevisiae*, a control sample (CK, without OP or FOP supplementation) was included to allow the functional effects of OP- and FOP-supplemented systems to be evaluated relative to a substrate-free baseline.

### 2.4 Non-targeted metabolomic profiling of OP and FOP

In the subsequent non-targeted metabolomic profiling, FOP or OP powder (1.0 g) underwent ultrasonic extraction for comparative compositional profiling, rather than quantitative yield assessment. The specific extraction procedure was as follows: An amount of 1.0 g of FOP or OP powder was weighed and added to 25 mL of 60% (v/v) high-performance liquid chromatography (HPLC)-grade ethanol solution, followed by ultrasonic extraction for 30 min. The extract was then subjected to centrifugation at 6000 rpm for 10 min. The supernatant obtained was diluted tenfold with methanol and filtered through a 0.22 μm membrane prior to LC/MS analysis.

Chromatographic analysis was conducted utilizing an ACQUITY HPLC I-Class system in conjunction with a Xevo G3-XS QTOF mass spectrometer (Waters, USA). The chromatographic separation was carried out on a Waters BEH T3 column (1.8 μm, 2.1 mm × 150 mm), which was maintained at a temperature of 40 °C. The mobile phase comprised (A) 0.1% formic acid in water and (B) acetonitrile, following a gradient elution program as follows: 0–1 min, 100% to 90% A; 1–3 min, 90% to 60% A; 3–10 min, 60% to 40% A; 10–20 min, 40% to 20% A; 20–27 min, 20% to 90% A; and 27–29 min, 90% A. The flow rate was maintained at 0.3 mL/min, with a detection wavelength set at 350 nm and an injection volume of 10 μL.

In the mass spectrometry analysis, an electrospray ionization (ESI) source was utilized in both positive and negative ionization modes, covering a mass range of 50–1200 Da. MSE data acquisition was conducted under the following conditions: a capillary voltage of 0.5 kV, an ion source temperature of 100 °C, a desolvation temperature of 400 °C, a desolvation gas flow rate of 800 L/h, a cone voltage of 50 V, and collision energies set at 4 V for low energy and 15–60 V for high energy. Data processing was performed using UNIFI software (version 1.9.2, Waters).

### 2.5 Alcohol fermentation experiment

In the alcoholic fermentation experiment, a fermentation medium (15 L) was prepared using deionized water and contained 250 g/L glucose, 1.5 g/L peptone, 1 g/L yeast extract, 1 g/L ammonium sulfate, 1.5 g/L potassium dihydrogen phosphate, and 0.65 g/L magnesium sulfate, as described by Comelli et al.(Comelli et al., 2016). Following sterilization, *S. cerevisiae* was inoculated at 5% (v/w) when the temperature decreased to 38 °C. The temperature was maintained for 30 min to facilitate activation, after which it was rapidly reduced to 28 °C. The experimental group was supplemented with 2 g/L FOP as a fermentation-derived product**,** to evaluate its potential influence on alcoholic fermentation performance. Samples were collected every 12 h to measure ethanol concentration and residual sugar content.

Determination of ethanol concentration: A distillation apparatus was assembled, and a mixture comprising 100 mL of the sample and 100 mL of deionized water was introduced into the distillation flask. The distillate, ranging from 90 to 100 mL, was collected and subsequently diluted to a total volume of 100 mL. The specific gravity of the diluted distillate was then determined using a hydrometer, as described by Spedding (Spedding, 2016).

Determination of residual sugar (Schwarz et al., 2020; Zhang et al., 2019): A 2 mL aliquot of the sample was transferred into a volumetric flask, to which 1.5 mL of DNS reagent was added. The mixture was subjected to heating in a boiling water bath for 5 min, followed by rapid cooling to room temperature using an ice-water bath. The volume was adjusted to 25 mL with deionized water. After thorough mixing, the absorbance was measured at a wavelength of 540 nm. The glucose standard curve was characterized by the linear regression equation y = 10.71× + 0.0169, with a correlation coefficient (R^2^) of 0.999.

### 2.6 Statistical analysis

All experiments were performed in triplicate to ensure data reliability, with each sample set comprising three replicates. Statistical analysis was conducted using IBM SPSS Statistics 27.0. Significance was evaluated by one-way analysis of variance (ANOVA), and Levene's test was used to assess the homogeneity of variance. When the assumption of homogeneity was met, the least significant difference (LSD) method was used for post-hoc multiple comparisons. In cases where variance homogeneity was not achieved, Duncan's method was applied instead. A significance threshold of *p* < 0.05 was established for all statistical comparisons. Data collection, recording, and visualization were conducted using Origin 2021 and GraphPad Prism 10.0.

## 3 Results and discussion

In this study, FOP was treated as an integrated fermentation product, containing both *Rhizopus*-modified orange peel components and fermentation-derived constituents. OP was used as an unfermented matrix control to evaluate the overall effect of fermentation treatment on the functional properties of orange peel-based products, rather than to establish a mass-equivalent comparison of orange peel components. For comparative evaluation, OP and FOP were added to the experimental systems on an equal mass basis to assess their overall functional effects. This comparison was designed to evaluate the impact of *Rhizopus* fermentation on the functional performance of orange peel–based products, rather than to achieve strict equivalence in the original orange peel content.

### 3.1 Optimization of FOP production

#### 3.1.1 Single-factor experiments

**Fig. 1:**
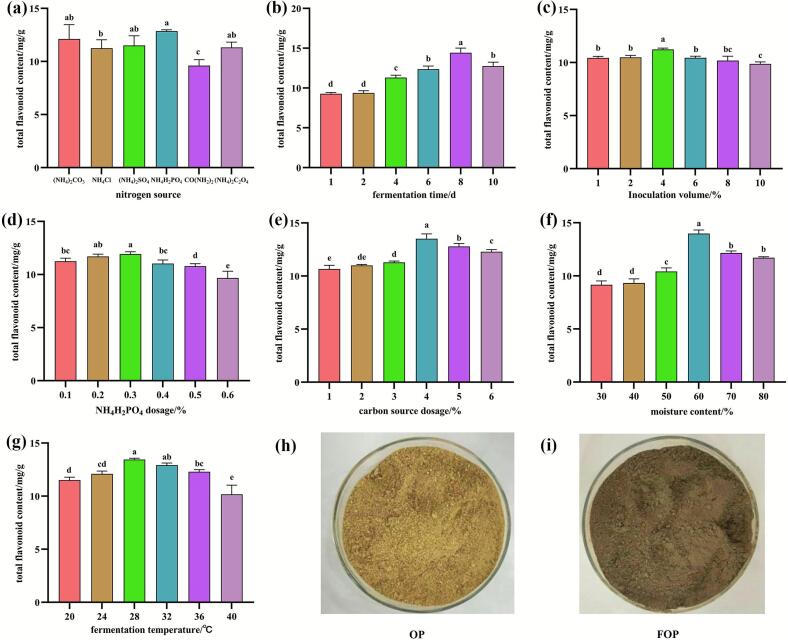
Effects of different factors on the total flavonoid content of FOP: a. nitrogen sources; b. fermentation time; c. inoculation volume; d. nitrogen source dosage; e. carbon source dosage; f. substrate moisture content; g. fermentation temperature. The appearance of: h. powder of OP; i. powder of FOP. Note: For panels a–g, bars with different lowercase letters (e.g., a, b, c) indicate significant differences (p < 0.05) among groups. Bars sharing no common letters (e.g., a vs. b) are significantly different, while those sharing at least one common letter (e.g., ab vs. a, ab vs. b) are not significantly different.

Fig. 1 demonstrates that the optimal fermentation period is 8 days (*p* < 0.05) with an inoculation volume of 4% (p < 0.05). The optimal nitrogen source is identified as 0.3% (w/w) ammonium dihydrogen phosphate (p < 0.05), while the optimal carbon source is 4% (w/w) glucose (p < 0.05), and the optimal moisture content is 60% (p < 0.05). Under these single-factor optimized fermentation conditions at 28 °C, the total flavonoid content in FOP reached 11.03 ± 0.18 mg/g DW (p < 0.05), which was higher than that measured in OP (9.85 ± 0.26 mg/g DW). These results indicate that *Rhizopus* fermentation was associated with an increased flavonoid level in the fermented orange peel system, providing a basis for further process optimization.

#### 3.1.2 RSM optimization

Based on the findings from the initial single-factor experiments, a Plackett-Burman experimental design consisting of 12 runs was implemented. The total flavonoid content served as the response variable. Analysis of the Plackett-Burman results revealed that moisture content (A), fermentation time (B), and NH_4_H_2_PO_4_ dosage (C) were the most significant factors, with a significance level of *p* < 0.05. Consequently, these factors were selected for further investigation through RSM. The experimental design and results of the RSM, aimed at optimizing the fermentation conditions of FOP, are detailed in Table 3.

**Table 3:** Response surface analysis of the design and results of FOP fermentation conditions.

Experiment number	A Moisture content (%)	B Fermentation time (d)	C NH_4_H_2_PO_4_ dosage (%)	Total flavonoid content (mg/g DW)
1	50	8	0.4	9.372
2	50	8	0.3	8.257
3	50	7	0.35	15.218
4	60	8	0.35	8.347
5	50	7	0.35	15.700
6	40	7	0.4	12.988
7	60	7	0.3	10.794
8	60	6	0.35	10.758
9	50	7	0.35	15.730
10	50	7	0.35	16.032
11	60	7	0.4	10.547
12	40	7	0.3	10.366
13	50	6	0.4	8.558
14	40	6	0.35	10.312
15	50	7	0.35	16.393
16	50	6	0.3	10.010
17	40	8	0.35	10.818

The experimental results were analyzed utilizing Design-Expert 13.0 through multiple quadratic regression analysis, resulting in the derivation of the regression equation for the total flavonoid content in FOP:

Y=15.8146−0.50475A−0.35550B+0.25475C−0.72925AB−0.71725AC+0.64175BCE−1.81568A2−3.94018B2−2.82517C2.

The analysis of variance (ANOVA) outcomes for the regression model was detailed in Table 4. The model demonstrated an F-value of 58.07 and a *p*-value of less than 0.01, indicating its statistical significance. Furthermore, the lack-of-fit *p*-value was 0.2830 (greater than 0.05), suggesting that the model adequately fits the experimental data. The coefficient of determination (R^2^ = 0.9868), along with the adjusted and predicted R^2^ values, were within acceptable ranges, underscoring the model's capability to effectively elucidate the influence of factor variations on the response and to predict optimal FOP fermentation conditions. Based on the F-values, the factors affecting the total flavonoid content in FOP were ranked as follows: moisture content (A) > fermentation time (B) > nitrogen source dosage (C). Among these, factor A exhibited significant effects (*p* < 0.05). The interaction terms AB, AC, and BC also demonstrated significant impacts (p < 0.05). The first-order terms A, B, and C, the interaction term BD, and the second-order terms B^2^ and C^2^ exhibited highly significant effects on the total flavonoid content (*p* < 0.01). These results affirm the suitability of the regression equation for determining the optimal fermentation conditions for FOP.

**Table 4:** Analysis of Variance Results for Response Surface Methodology of Fermentation Process.

Source	Sum of Squares	df	Mean Square	F-value	p-value	Significance
Model	133.94	9	14.88	58.07	<0.0001	**
A	2.04	1	2.04	7.95	0.0258	*
B	1.01	1	1.01	3.94	0.0874	
C	0.5192	1	0.5192	2.03	0.1977	
AB	2.13	1	2.13	8.30	0.0236	*
AC	2.06	1	2.06	8.03	0.0253	*
BC	1.65	1	1.65	6.43	0.0389	*
A^2^	13.88	1	13.88	54.16	0.0002	
B^2^	65.37	1	65.37	255.05	<0.0001	**
C^2^	33.61	1	33.61	131.13	<0.0001	**
Residual	1.79	7	0.2563			
Lack of Fit	1.04	3	0.3453	1.82	0.2830	
Pure Error	0.7580	4	0.1895			
Cor Total	135.73	16				
R^2^			0.9868	Pred R^2^	0.8691	
Adj R^2^			0.9698	Adeq Precisior	20.6484	

Note: Statistical significance is denoted as p < 0.01 (highly significant) and p < 0.05 (significant), where asterisks (**) and (*) indicate the respective significance levels.

**Fig. 2:**
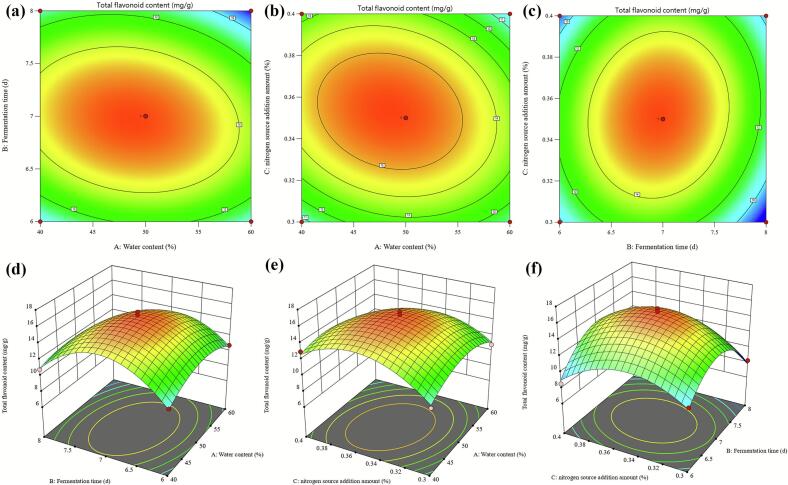
Contour and response surface plots illustrating the interaction of various factors on total flavonoid content: a. Contour plots of the interaction between fermentation time and moisture content; b. Contour plots of the interaction between nitrogen source dosage and moisture content; c. Contour plots of the interaction between fermentation time and nitrogen source dosage; d. Response surface plots of the interaction between fermentation time and moisture content; e. Response surface plots of the interaction between nitrogen source dosage and moisture content; f. Response surface plots of the interaction between fermentation time and nitrogen source dosage.

An analysis of contour plots and response surface gradients indicated that steeper slopes were associated with more pronounced impacts on the total flavonoid content in FOP. Variations in response values, resulting from changes in fermentation parameters, demonstrated both the sensitivity and statistical significance of factor interactions. The strength of these interactions was visually discernible through contour geometry: elliptical patterns (aspect ratio > 1.5:1) or slopes exceeding 35° indicated strong interactions, whereas near-circular contours (aspect ratio < 1.2:1) were indicative of weaker effects. Fig. 2 presents the contour and response surface plots that illustrate the effects of factor interactions on total flavonoid content.

The regression model identified optimal fermentation conditions as follows: a moisture content of 48.54%, a fermentation duration of 6.97 days, and a nitrogen source dosage of 0.35%, resulting in a predicted total flavonoid content of 15.86 mg/g DW. When adjusted for practical experimental application, the parameters were modified to a water content of 50%, a fermentation duration of 7 days, and a nitrogen source dosage of 0.35%. Under these optimized conditions, the actual total flavonoid content in FOP was measured at 15.74 ± 0.06 mg/g DW. In comparison, the total flavonoid content in unfermented orange peel was 9.85 ± 0.26 mg/g DW. This difference reflects a pronounced alteration in flavonoid levels between the fermented and unfermented orange peel systems. Moreover, RSM optimization resulted in a higher flavonoid content in FOP compared to non-optimized fermentation conditions (11.03 ± 0.18 mg/g DW), indicating that fermentation parameters strongly influence the compositional profile of the fermented product.

### 3.2 Impact of FOP and OP on growth and stress tolerance in *S. cerevisiae*

**Fig. 3:**
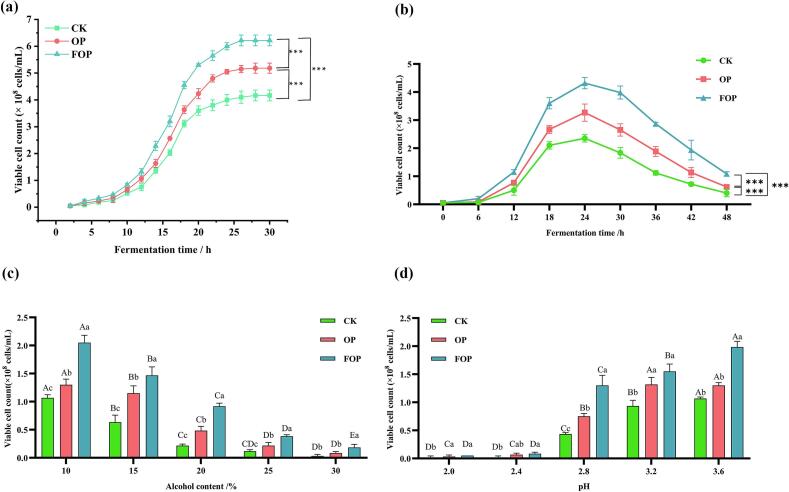
Effects of FOP and OP on growth and stress tolerance: a. growth of *S. cerevisiae*; b. viability of cells under thermal stress (40 °C); c. viability of cells under higher alcohol concentration stress; d. viability of cells under lower pH stress conditions. *Note: The* symbol “***” denotes an extremely significant difference in comparison to the control group (p < 0.001). In panels a-d, bars marked with distinct lowercase letters represent significant differences within groups, whereas bars marked with distinct uppercase letters signify significant differences between groups (*p* < 0.05). Groups or levels that do not share any common letters (e.g., A vs. B, a vs. b) are considered significantly different, while those that share at least one common letter (e.g., AB vs. A, ab vs. a) are not considered significantly different.

As illustrated in Fig. 3a, both OP and FOP treatments substantially promoted the growth of *S. cerevisiae*. Notably, within the 2- to 4-h interval, microbial biomass in the CK, OP, and FOP groups increased from 0.05 × 10^8^ to 0.08 × 10^8^, 0.15 × 10^8^, and 0.22 × 10^8^ cells/mL, respectively. The specific growth rate (μ) of the FOP group during this period was 3.09 times greater than that of the CK group and 1.42 times greater than that of the OP group, underscoring the stronger growth-promoting effect observed in the FOP-supplemented system compared with the OP-supplemented and control groups under the conditions tested. At the stationary phase, the FOP group demonstrated significantly higher microbial biomass, exhibiting increases of 20.01% and 49.44% relative to the OP and CK groups, respectively (Fig. 3a). These differences were statistically significant (*p* < 0.001). In summary, FOP exhibits considerable potential in enhancing yeast growth and proliferation dynamics. These observations suggest that FOP supplementation may influence yeast growth dynamics, which could be relevant for fermentation performance under specific conditions.

#### 3.2.1 Thermal stress

During fermentation, microbial growth generates considerable metabolic heat, necessitating substantial energy expenditure for cooling, which constitutes a significant portion of production costs. A prevalent industry approach involves screening for thermotolerant strains or employing additives to enhance heat resistance. In experiments where *S. cerevisiae* was subjected to 40 °C for various durations, systems supplemented with FOP demonstrated higher viable cell counts compared to the CK groups. After 24 h of culture at 40 °C, the viable cell counts were 2.35 × 10^8^ cells/mL in the CK group, 3.08 × 10^8^ cells/mL in the OP group, and 4.32 × 10^8^ cells/mL in the FOP group (Fig. 3b). Given that the optimal growth temperature for *S. cerevisiae* is typically 28 °C, the use of FOP shows significant potential for facilitating high-temperature fermentation, thereby offering promising prospects for industrial applications.

#### 3.2.2 High ethanol concentrations

Elevated ethanol concentrations were found to be detrimental to the growth of *S. cerevisiae*, with inhibition typically occurring at concentrations exceeding 10%. Experimental findings indicated that following a 2-h exposure to ethanol concentrations between 10% and 30%, the viable cell count in the FOP-supplemented system was markedly higher than that observed in the CK group (refer to Fig. 3c). Notably, at a 20% ethanol concentration, the viable yeast cell count in the FOP group was 0.92 × 10^8^ cells/mL, representing an increase of 4.23-fold compared to the CK group and 2.39-fold compared to the OP group. At a 30% ethanol concentration, the viable cell count in the CK group was 0.03 × 10^8^ cells/mL, whereas the OP group exhibited a count of 0.08 × 10^8^ cells/mL. In contrast, the FOP group demonstrated a viable cell count of 0.18 × 10^8^ cells/mL, which was 6.0 times greater than that of the CK group and 2.25 times greater than that of the OP group.

#### 3.2.3 Low-pH conditions

In the context of fermentation, a low-pH environment effectively inhibits the proliferation of various microorganisms, thereby preventing contamination. However, elevated concentrations of hydrogen ions also impede yeast growth. Consequently, acid tolerance is essential for optimal yeast fermentation. Fig. 3d demonstrates that low pH conditions markedly inhibit yeast growth, with pronounced suppression occurring at pH levels below 2.8 (Cui et al., 2025). At a pH of 2.8, yeast growth remained significantly inhibited in the control (CK) group; nevertheless, the viable cell counts in samples supplemented with FOP reached 1.30 × 10^8^ cells/mL, which was 3.02 times higher than that of the CK group and 1.73 times greater than that of the OP group. While *S. cerevisiae* typically exhibits substantially inhibited growth below pH 4.0, FOP supplementation extended its acid tolerance threshold to pH 2.8–3.2, illustrating its remarkable capacity to enhance yeast's resistance to acid stress (Fig. 3d).

### 3.3 Non-targeted metabolomic profiling of FOP

**Fig. 4:**
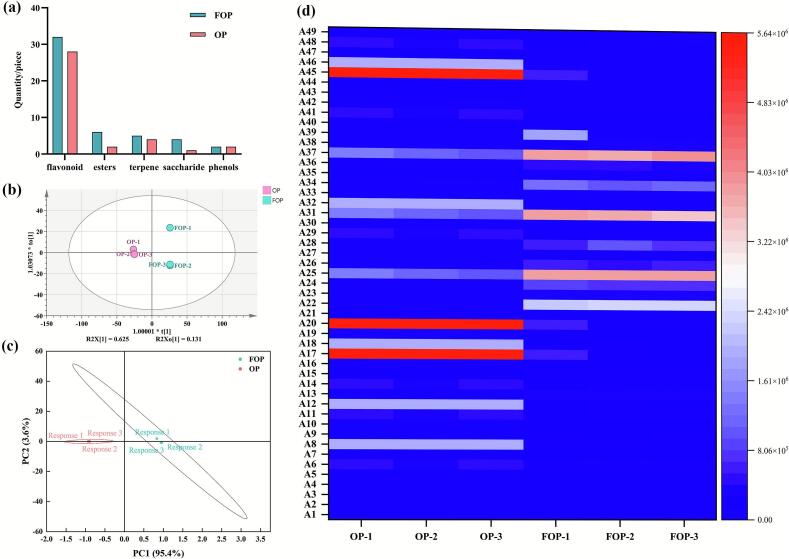
Differences between OP and FOP samples: a. Category difference map between OP and FOP samples (visualizing the classification of differential metabolites); b. OPLS-DA score plot of OP and FOP samples (showing the supervised discriminant analysis between groups, R^2^Y = 1, Q^2^ = 0.991); c. PCA score plot of OP and FOP samples (unsupervised clustering analysis, showing cumulative variance contribution rate PC1 = 95.4%, PC2 = 3.6%); d. Heatmap of 49 key components in OP and FOP (hierarchical clustering based on Euclidean distance, with red indicating higher relative content and blue indicating lower content compared to the median value across all samples). (For interpretation of the references to colour in this figure legend, the reader is referred to the web version of this article.)

Non-targeted metabolomic profiling was employed to systematically compare the chemical compositions of OP and FOP. In total, 1045 metabolites were detected in OP, whereas 1299 metabolites were identified in FOP, indicating that fermentation substantially increased the chemical complexity of the orange peel matrix. Among these metabolites, 254 compounds were uniquely detected or significantly enriched in FOP. Multivariate statistical analyses further confirmed the marked compositional differences between OP and FOP (Fig. 4). The OPLS-DA model exhibited strong discriminative power with excellent model fitness and predictability (R^2^Y = 1.000, Q^2^ = 0.991), and permutation testing excluded overfitting, supporting the robustness of the analysis. In addition, PCA score plots clearly demonstrated distinct clustering between OP and FOP samples, reflecting substantial metabolic divergence induced by fermentation. Based on the selection criteria of VIP ≥ 1, fold change (FC) ≥ 2 or ≤ 0.5, and *p* < 0.05, a total of 49 differential metabolites were identified as key contributors to the compositional differences between OP and FOP (Table 5).

**Table 5:** Comparative analysis of 49 constituent compounds between OP and FOP.

Number	Name	OP relative abundance/%	FOP relative abundance/%	Material category
A1	(+)-15-Methoxyfloridiolide A	ND	0.64 ± 0.06^gh^	Esters
A2	(2E,4Z,6Z,8E,10E,12E,14Z,16Z,18E)-1,20-Bis[(1*R*,4*S*)-4-hydroxy-1,2,2-trimethylcyclopentyl]-4,8,13,17-tetramethylicosa-2,4,6,8,10,1	ND	0.72 ± 0.07^gh^	Esters
A3	(2E,8E)-2,8-Decadiene-4,6-diyne-1,10-diol 1-O-beta-D-glucopyranoside	ND	0.87 ± 0.08^fgh^	Saccharides
A4	(4e)-1,5-Bis(4-hydroxyphenyl)-2-(methoxymethyl)-4-penten-1-ol	0.00 ± 0.00^h^	0.69 ± 0.05^gh^	Flavonoid
A5	(5*R*)-5-Ethoxy-7-(4-hydroxy-3-methoxyphenyl)-1-phenylheptan-3-one	ND	0.809 ± 0.10^gh^	Flavonoid
A6	1,5-Bis(4-hydroxy-3-methoxyphenyl)-1,4-pentadien-3-one	1.20 ± 0.03^d^	0.15 ± 0.02^h^	Flavonoid
A7	1-Oxomiltirone	0.14 ± 0.00^gh^	1.02 ± 0.10^fgh^	Terpene
A8	2-(4-Hydroxyphenethyl)-6-hydroxychromone	5.68 ± 0.07^b^	0.24 ± 0.32^h^	Flavonoid
A9	21-(2-Oxopropyl) koumine	ND	0.57 ± 0.12^gh^	Terpene
A10	2’-Hydroxymethylophiopogonone A	ND	0.72 ± 0.08^gh^	Flavonoid
A11	3′,4′,5′,5,7-Pentamethoxyflavone	1.20 ± 0.03^d^	0.20 ± 0.22^h^	Flavonoid
A12	3′,4’-Dimethoxyflavone	5.688 ± 0.07^b^	0.49 ± 0.32^gh^	Flavonoid
A13	3,4-Dihydroxyflavone	0.25 ± 0.01^fgh^	1.33 ± 0.13^efgh^	Flavonoid
A14	3,5,7,3′,4′-Pentamethoxyflavone	1.20 ± 0.03^d^	0.20 ± 0.22^h^	Flavonoid
A15	3,5-di-O-Caffeoyl-4-O-(3-hydroxy-3-methyl) glutaroylquinic acid	0.71 ± 0.01^e^	0.10 ± 0.06^h^	Esters
A16	3,6-Di(4-hydroxy) benzyl-2,5-dioxopiperazine	ND	0.95 ± 0.15^fgh^	Flavonoid
A17	3-Acetylpinobanksin-7-methyl ether	16.26 ± 0.18^a^	1.08 ± 1.07^efgh^	Flavonoid
A18	5,7-Dimethoxyflavone	5.68 ± 0.07^b^	0.49 ± 0.32^gh^	Flavonoid
A19	5-Methoxydehydrodiisoeugenol	0.14 ± 0.00^gh^	1.01 ± 0.09^fgh^	Phenols
A20	5-*O*-Demethylnobiletin	16.26 ± 0.18^a^	0.89 ± 1.17^fgh^	Flavonoid
A21	7-O-Methylwogonin 5-glucoside	0.69 ± 0.00^e^	0.33 ± 0.03^gh^	Flavonoid
A22	Acacetin 7-O-(6′′-O-α-L-rhamnopyranosyl -β- sophoroside)	0.01 ± 0.00^h^	10.05 ± 1.06^b^	Flavonoid
A23	Apigenin-7-O-(2G-rhamnosyl) gentiobioside	0.04 ± 0.00^h^	1.68 ± 0.14^efgh^	Flavonoid
A24	Benzpinacol	0.07 ± 0.00^h^	3.35 ± 0.27^d^	Phenols
A25	Cusianoside A	3.33 ± 0.38^c^	16.82 ± 1.44^a^	Flavonoid
A26	*D* – (+) - Trehalose	0.04 ± 0.00^h^	2.38 ± 0.15^def^	Saccharides
A27	Daechuine S 7	0.08 ± 0.01^h^	0.94 ± 0.08^fgh^	Terpene
A28	D-Mannuronic acid	ND	3.52 ± 1.16^d^	Saccharides
A29	Eucalyptin	1.20 ± 0.03^d^	0.27 ± 0.17^gh^	Flavonoid
A30	Gypenoside XXIX	0.10 ± 0.00^h^	0.97 ± 0.076^fgh^	Flavonoid
A31	Hesperidin	3.33 ± 0.38^c^	16.06 ± 1.12^a^	Flavonoid
A32	Isobonducellin	5.68 ± 0.07^b^	0.49 ± 0.33^gh^	Flavonoid
A33	Kuwanon K	0.36 ± 0.02^fg^	0.03 ± 0.01^h^	Flavonoid
A34	L-Arabinose	ND	4.97 ± 0.22^c^	Saccharides
A35	Lethedoside A	0.69 ± 0.00^e^	0.11 ± 0.12^h^	Flavonoid
A36	Methyl Arjunolate	ND	1.88 ± 0.31^efg^	Esters
A37	Neohesperdin	3.33 ± 0.38^c^	16.78 ± 1.42^a^	Flavonoid
A38	Neoisorutin	ND	1.44 ± 0.39^efgh^	Flavonoid
A39	Octadecadienoic acid	ND	2.37 ± 3.25^de^	Esters
A40	Phanoside	0.39 ± 0.01^f^	0.02 ± 0.01^h^	Esters
A41	Pilloin	1.20 ± 0.03^d^	0.59 ± 0.05^gh^	Flavonoid
A42	Rutin	0.03 ± 0.00^h^	0.37 ± 0.25^gh^	Flavonoid
A43	Saikosaponin T	0.44 ± 0.16^f^	0.02 ± 0.00^h^	Terpene
A44	Scroneoside A	0.69 ± 0.00^e^	0.11 ± 0.13^h^	Terpene
A45	Scutellarein tetramethyl ether	16.26 ± 0.18^a^	0.92 ± 1.15^fgh^	Flavonoid
A46	Tanshinone VI	5.68 ± 0.07^b^	0.11 ± 0.00^h^	Flavonoid
A47	Vitexin arginine	0.45 ± 0.01^f^	0.05 ± 0.04^h^	Flavonoid
A48	Hesperetin	1.20 ± 0.03^d^	0.20 ± 0.22^h^	Flavonoid
A49	Mulberry flavone G	0.36 ± 0.02^fg^	0.03 ± 0.01^h^	Flavonoid

Note: ND indicates that the compound was not detected in the corresponding sample.

Classification analysis revealed that these differential metabolites mainly belonged to flavonoids, saccharides, esters, terpenoids, and phenolic compounds. Notably, fermentation resulted in an overall enrichment of flavonoids and saccharides, which are widely reported to be associated with antioxidant activity and cellular stress adaptation in various biological systems. Among the differential metabolites, several flavonoids showed pronounced increases in relative abundance after fermentation. In particular, hesperidin, neohesperidin, and rutin were identified as representative compounds due to their relatively high abundance and significant enrichment in FOP. Hesperidin and neohesperidin accounted for more than 16% of the relative abundance each in FOP, representing approximately a fivefold increase compared to OP. In addition, the content of rutin exhibited an approximate 11.7-fold increase after fermentation.

**Fig. 5:**
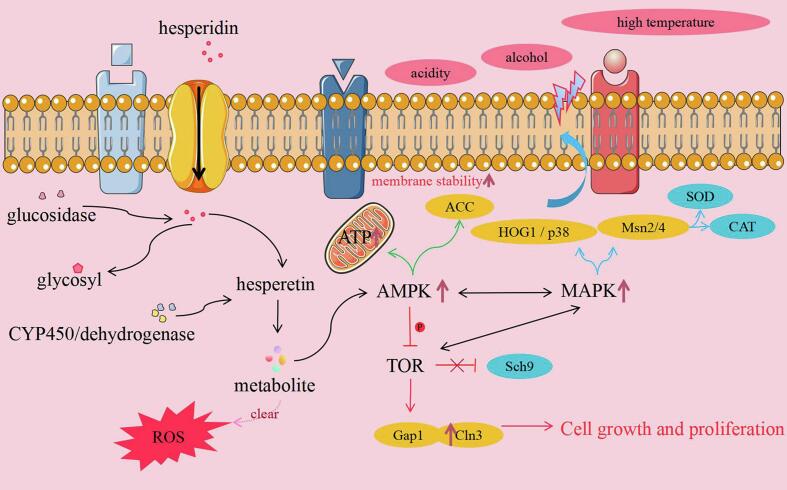
Intracellular metabolic pathway of hesperidin.

Previous studies have reported that these flavonoids (e.g., hesperidin, neohesperidin, rutin) possess inherent antioxidant and cytoprotective properties, which have attracted extensive attention; these compounds undergo dynamic metabolic changes during fermentation and exert protective effects in *S. cerevisiae* and other eukaryotic models (Liang et al., 2023). Hesperidin is a citrus flavonoid glycoside that is widely distributed in Citrus plants (Yuping Li et al., 2024). It facilitates cell proliferation and division by modulating the expression of proteins related to the cell cycle, while its antioxidant properties alleviate oxidative stress, thereby fostering a healthier microenvironment for cellular activities. During yeast growth, hesperidin is transported into yeast cells via membrane transporters and subsequently hydrolyzed by glycosidases to produce hesperetin and a glycosyl moiety. Hesperetin, as the active form, undergoes further modifications such as hydroxylation, methylation, or oxidation, mediated by cytochrome P450 enzymes or dehydrogenases (Choi et al., 2022; Roohbakhsh et al., 2014), resulting in the formation of 3′-hydroxyhesperetin, 7-*O*-methylhesperetin, hesperetin quinone, and hesperetin glucuronide—metabolites that effectively scavenge intracellular ROS and demonstrate significant antioxidant activity (Lee et al., 2012; Rizza et al., 2011). Furthermore, hesperidin and its metabolites enhance the efficiency of ATP production by regulating glycolysis and the mitochondrial respiratory chain, and may activate the AMPK energy sensor pathway to optimize energy metabolism (Martínez-Noguera et al., 2020; Ohtsuka et al., 2024; Shokri Afra et al., 2019). By modulating the MAPK and TOR signaling pathways, hesperidin influences cellular stress responses, improves membrane permeability, and promotes the uptake and utilization of glucose and amino acids (Fig. 5) (Alugoju et al., 2023). Collectively, hesperidin enhances yeast tolerance through multifaceted mechanisms, including antioxidant activity, regulation of energy metabolism, modulation of signaling pathways, and promotion of nutrient absorption. As a flavonoid compound structurally similar to hesperidin, neohesperidin has been found in studies to possess anti-aging potential and can extend the physiological lifespan of *S. cerevisiae* (Lin et al., 2021). Compared with rutin, naringenin, and 3,4-dihydroxyflavone, neohesperidin exhibits the most significant lifespan-extending effect on *S. cerevisiae* at a certain concentration. Additionally, the flavonoids quercetin and apigenin activate metabolic pathways in yeast cells, facilitating sugar breakdown and energy production to enhance yeast metabolic activity (Ali et al., 2016).

Quercetin is the aglycone of rutin. Studies have shown that pretreatment with quercetin at low concentrations (10 μM and 100 μM) can enhance the viability of *S. cerevisiae* upon exposure to hydrogen peroxide, copper ions, and heat shock, with these stress-protective effects associated with the alleviation of oxidative stress but not limited to antioxidant activity—they may also involve other cytoprotective mechanisms(de Noronha et al., 2022). Rutin augments yeast tolerance via its metabolic pathway: within yeast cells, it is initially hydrolyzed by glycosidases to yield quercetin and a glycosyl moiety, and quercetin then undergoes hydroxylation, methylation, or oxidation to produce various metabolites with significant antioxidant properties (Lin et al., 2022; Zhou et al., 2025). A common mechanism of these flavonoids involves alleviating oxidative stress: their metabolites effectively scavenge intracellular ROS, modulate glutathione levels, and enhance the activity of antioxidant enzymes such as SOD and CAT (Chen et al., 2022; Li et al., 2022; Wu, Zhang, et al., 2024). Additionally, rutin and its metabolites may improve yeast tolerance to thermal and osmotic stress by activating heat shock proteins (HSPs) and regulating the synthesis of osmoprotectants such as trehalose and glycerol, thereby significantly enhancing yeast resilience to a variety of environmental stressors (Abomosallam et al., 2024; Chen et al., 2022).

Therefore, the enrichment of these flavonoids in FOP may be associated with improved cellular redox balance, membrane stability, and overall stress adaptability in *S. cerevisiae*. It should be emphasized that the present study did not conduct direct experimental validation of key indicators (e.g., intracellular ROS levels, antioxidant enzyme activities, metabolic flux, membrane composition). Thus, the biological relevance of these metabolites (flavonoids, saccharides, fatty acids) to yeast stress tolerance is discussed in a correlative and speculative context-supported by metabolomic data and prior literature-rather than as experimentally confirmed causal mechanisms.

In addition to flavonoids, several saccharides and fatty acid–related metabolites were newly detected or significantly enriched in FOP, including L-arabinose, D-mannuronic acid, and octadecadienoic acid. These compounds may be metabolically relevant to yeast stress responses through their potential involvement in energy metabolism, redox homeostasis, and membrane composition. For instance, L-arabinose can enter pentose phosphate–related metabolic routes, which are known to generate NADPH and support cellular antioxidant capacity, while unsaturated fatty acids such as octadecadienoic acid may influence membrane fluidity and integrity.

Taken together, metabolomic data indicate the enrichment of multiple bioactive compounds in FOP. Synergistic effects contribute significantly to the enhanced yeast stress tolerance: neohesperidin acts synergistically with other citrus flavonoids to strengthen anti-aging activity, and collectively, these fermentation-enriched metabolites (rather than any single compound) function through non-exclusive effects to improve *S. cerevisiae*'s growth performance and stress adaptability.

Overall, this study provides a metabolomic basis for understanding the potential contribution of FOP to yeast stress tolerance. However, further targeted investigations, such as single-compound supplementation, transcriptomic analysis, or intracellular redox measurements, will be necessary to verify the specific molecular mechanisms underlying these effects.

### 3.4 Results of the alcoholic fermentation experiment

**Fig. 6:**
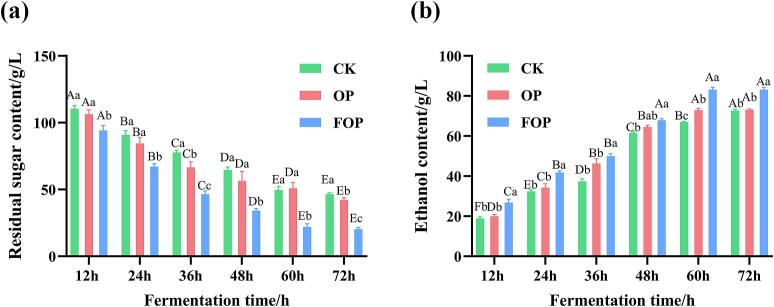
Effect of OP and FOP on the alcohol fermentation: a. Residual sugar content; b. Ethanol content. *Note:* Same as above.

We conducted a further investigation into the influence of FOP on alcohol fermentation within a 20 - l fermenter. Fig. 6(a) shows a gradual decline in residual sugar levels across all three systems over time. From 12 h to 72 h, sugar content dropped from 110.38 g/L, 106.33 g/L, and 94.19 g/L to 46.41 g/L, 42.13 g/L, and 20.26 g/L for CK, OP, and FOP systems, respectively. Notably, during the final fermentation stage (60–72 h), the FOP system had the lowest sugar level at 20.26 g/L, which was 56.3% and 51.9% less than the CK and OP systems.

Fig. 6(b) illustrates that ethanol levels rose over time in all systems, with the FOP-supplemented system consistently showing higher concentrations. The FOP system's ethanol levels reached 83.10 g/L by 72 h, peaking with a 41.80% relative increase at 12 h and a 16.17 g/L absolute increase at 60 h compared to the CK control. By the end of fermentation, the FOP system's ethanol concentration was 10.50 g/L higher than the CK group's, marking a 14.46% improvement. These results align with typical ethanol production ranges in similar fermentation studies (Huang et al., 2020; Sugiyama et al., 2018).

These results indicate that the addition of FOP may significantly improve fermentation performance by promoting both sugar consumption efficiency (with 56.3% lower residual sugar at 72 h) and ethanol production capacity (with an average 28.94% relative increase in ethanol concentration across the entire fermentation period), although the specific contribution of individual fermentation-derived components cannot be distinguished.

## 4 Conclusion

This study illustrates that the optimized fermentation of orange peel by *R. stolonifer* produces a bioactive-rich product (FOP) that markedly enhances the performance of *S. cerevisiae*. Through systematic process optimization, the bioactive components in FOP are effectively enriched, significantly improving yeast tolerance to various industrially relevant stresses, such as ethanol, temperature, and acidity, while simultaneously promoting growth and viability. Metabolomic analysis indicates that the efficacy of FOP is attributed to its enriched composition of flavonoids, esters, terpenoids, saccharides, and phenols, which collectively enhance yeast physiology through synergistic antioxidant, metabolic, and stress-protective mechanisms. This innovative approach not only offers an effective strategy for augmenting industrial yeast performance but also establishes a sustainable valorization pathway for citrus processing byproducts, with particular significance for bioethanol production. In 15-l ethanol fermentation experiments, the incorporation of FOP was observed to enhance the alcohol content. Future research endeavors will focus on examining the long-term stability of the bioactive components in FOP during storage, as well as investigating its potential synergistic interactions with other natural additives to further optimize yeast-based fermentation processes. It should be noted that the mass-based comparison between OP and FOP does not represent a strict equivalence in the original orange peel content, as FOP also contains fermentation-derived biomass and medium residues. Therefore, the observed differences reflect the overall functional outcome of *Rhizopus*-mediated fermentation, rather than a quantitative yield comparison of orange peel–derived bioactive compounds. In this context, OP served as a substrate-matched control, while FOP represented the corresponding fermentation-modified product.

## CRediT authorship contribution statement

**Xinjie Wang:** Writing – review & editing, Writing – original draft, Visualization, Methodology, Investigation, Formal analysis, Data curation, Conceptualization. **Tao Chen:** Writing – review & editing, Writing – original draft, Visualization, Methodology, Investigation, Formal analysis, Data curation, Conceptualization. **Dan Yu:** Writing – review & editing, Writing – original draft, Supervision, Software, Resources, Methodology, Investigation, Data curation. **Jinping Li:** Methodology, Investigation, Formal analysis, Data curation. **Yang Zhang:** Supervision, Resources, Project administration, Funding acquisition. **Jianxing Yu:** Writing – review & editing, Writing – original draft, Supervision, Resources, Project administration, Methodology, Investigation, Funding acquisition, Conceptualization. **Jiayou Li:** Writing – review & editing, Writing – original draft, Supervision, Resources, Project administration, Methodology, Investigation, Funding acquisition, Conceptualization.

## Declaration of competing interest

The authors declare that they have no known competing financial interests or personal relationships that could have appeared to influence the work reported in this paper.

## Data Availability

Data will be made available on request.
